# Suppression of osteopontin inhibits chemically induced hepatic carcinogenesis by induction of apoptosis in mice

**DOI:** 10.18632/oncotarget.13529

**Published:** 2016-11-23

**Authors:** Su-Hyung Lee, Jun-Won Park, Sang-Ho Woo, Du-Min Go, Hyo-Jung Kwon, Ja-June Jang, Dae-Yong Kim

**Affiliations:** ^1^ Department of Veterinary Pathology, College of Veterinary Medicine, Seoul National University, Seoul 151-742, South Korea; ^2^ Biomolecular Function Research Branch, National Cancer Center, Goyang, Gyeonggi 410-769, South Korea; ^3^ Department of Veterinary Pathology, College of Veterinary Medicine, Chungnam National University, Daejeon 305-764, South Korea; ^4^ Department of Pathology, College of Medicine, Seoul National University, Seoul 110-799, Korea

**Keywords:** osteopontin, EGFR, c-Jun, apoptosis, hepatocellular carcinoma

## Abstract

Previous clinical reports have found elevated osteopontin (OPN) levels in tumor tissues to be indicative of greater malignancy in human hepatocellular carcinoma (HCC). However, the role of OPN on carcinogenesis and its underlying mechanism remain unclear. In the present study, we investigated the oncogenic role of OPN in diethylnitrosamine (DEN)-induced hepatic carcinogenesis in mice. The overall incidence of hepatic tumors at 36 weeks was significantly lower in OPN knockout (KO) mice than in wild-type (WT) mice. Apoptosis was significantly enhanced in OPN KO mice, and was accompanied by the downregulation of epidermal growth factor receptor (EGFR). In the *in vitro* study, OPN suppression also led to lower mRNA and protein levels of EGFR associated with the downregulation of c-Jun in Hep3B and Huh7 human HCC cells lines, which resulted in increased apoptotic cell death in both cell lines. Moreover, a positive correlation was clearly identified between the expression of OPN and EGFR in human HCC tissues. These data demonstrate that the OPN deficiency reduced the incidence of chemically induced HCC by suppressing EGFR-mediated anti-apoptotic signaling. An important implication of our findings is that OPN positively contributes to hepatic carcinogenesis.

## INTRODUCTION

Liver cancer is the second most common cause of cancer-related death, and its incidence and mortality are prominent in East Asia, including Korea, Japan, and China [1]. Many studies have determined that various etiologies, including chronic hepatitis B or C viral infection and cirrhosis are important risk factors for HCC [2, 3]. Each factor or combination of these factors can give rise to an inflammatory response and DNA damage, which progress through chronic hepatitis, cirrhosis, and eventually HCC [4–6]. Nagoshi established that HCC-associated liver disease were strongly associated with the expression of OPN in various cells, including hepatocytes, Kupffer cells, and stellate cells [7].

OPN is a secreted glycophosphoprotein that acts a ligand for its receptors, including integrins and CD44 variants, and the interactions between OPN and its receptors promote a variety of signaling pathways that eventually result in tumor progression [8]. Clinical reports have found that a range of tumor tissues showed higher OPN expression than adjacent normal tissue [9, 10]. Likewise, it is accepted that OPN plays a crucial role in the oncogenesis of HCC, and that OPN overexpression is positively correlated with tumor progression [7]. Zhang *et al*. established that the binding of OPN to integrin and CD44 activated MMP-2, resulting in greater invasiveness [11]. In addition, HCC patients displaying the elevated expression of OPN mRNA in tumor tissues had a higher risk of intrahepatic metastasis and early recurrence [12]. Based on above findings, it is reasonable to conclude that OPN is a candidate biomarker and target for HCC therapy.

EGFR is a transmembrane growth factor regulated by receptor dimerization, which could be transactivated by OPN. The transactivation of EGFR principally upregulates the mitogen-activated protein kinase (MAPK), signal transducer and activator of transcirption (STAT) 3 and phosphoinositide 3-kinase/Akt signaling pathways [13, 14]. Although EGFR is essential in the regulation of normal development and cell differentiation, it has been proposed that EGFR activation may be tightly linked with the carcinogenesis of solid tumors [15–17]. Moreover, Harada *et al*. found that cirrhotic liver tissue and HCC tumor tissue tended to show EGFR overexpression [18].

Over the past decades, several reports have demonstrated that OPN overexpression in tumor tissues indicate more advanced tumor stages in human HCC, and that OPN is a viable marker for determining the prognosis, in combination with other factors [19–21]. However, the role of OPN on tumor development and the underlying mechanism remain poorly understood. In this study, we assessed how OPN deficiency affected liver carcinogenesis *in vivo* through a DEN-induced mouse HCC model and *in vitro* using human HCC cell lines, with the result that OPN was overexpressed in the tumor tissue in human HCC samples. We found that OPN played an oncogenic role in DEN-induced hepatic carcinogenesis, accompanied by the upregulation of EGFR.

## RESULTS

### Lack of OPN suppresses DEN-induced hepatic carcinogenesis

Macroscopically, the nodules displayed protruding single-to-multiple polypoid patterns in both the WT and OPN KO mice at 36 weeks after DEN injection. The properties of the nodules are summarized in Table 1. The size of the nodules in the OPN KO mice (1.2 ± 0.2 mm) was significantly smaller than that of WT mice (7.3 ± 1.8 mm; *P*<0.01), although the multiplicity was not significantly different between OPN KO mice (3.9 ± 0.9) and the WT mice (4.9 ± 1.3).

**Table 1:** Incidence and multiplicity of DEN-induced liver tumors in mice

OPN genotype	DEN treat	Weeksafter DEN	Mice (n)	Tumor-bearingmice (%)	Hepatocellular adenoma (%)	Hepatocellular carcinoma (%)	Tumor multiplicity	Tumor size diameter (mm)
WT	Yes	26	14	0	0	0	0	0
KO	Yes	26	15	2/15 (13.3)	2/15 (13.3)	0	1.7 ± 0.5	0.8 ± 0.3
WT	No	26	7	0	0	0	0	0
KO	No	26	7	0	0	0	0	0
WT	Yes	36	13	8/13 (61.5)	8/13 (61.5)	2/13 (15.4)	4.9 ± 1.3	7.3 ± 1.8
KO	Yes	36	14	2/14 (14.3) *	2/14 (14.3)	0	3.9 ± 0.9	1.2 ± 0.2 **
WT	No	36	7	0	0	0	0	0
KO	No	36	7	0	0	0	0	0

Data are presented as means±SEMs.

*P < 0.05

**P < 0.01 versus WT mice.

OPN, osteopontin; DEN, diethylnitrosamine; WT, wild-type; KO, knockout

**Figure 1:**
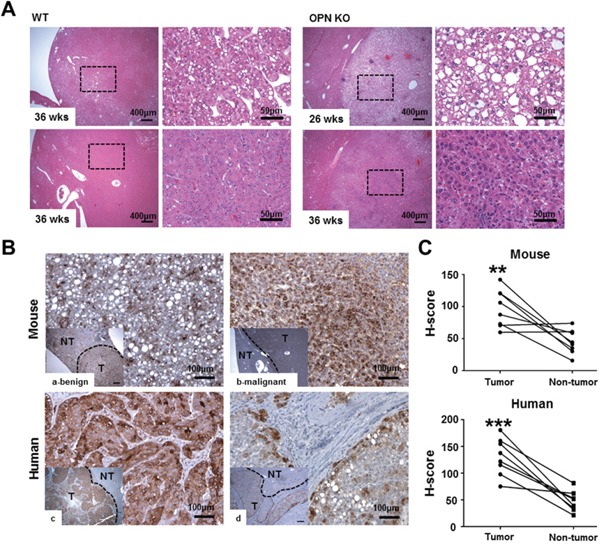
Histopathology and IHC for OPN **A**. Hematoxylin and eosin staining in liver tissue samples from tumor-bearing WT and OPN KO mice. The boxed regions of the left panels in the WT and OPN KO samples are shown at higher magnification in the right panels. The nodules from the OPN KO mice at 26 and 36 weeks were sharply circumscribed and composed of well-differentiated tumor cells or vacuolated cells that formed trabeculae or nests, whereas the nodules from the WT mice at 36 weeks showed a sessile and solid growth pattern. **B, C**. IHC and quantification for OPN in mouse and human liver tissue samples. The tumor tissue samples (T) showed greater cytoplasmic expression of OPN than non-tumor tissue sample (NT) in both mouse (a, b) and human (c, d) samples. In mice, hepatocellular carcinoma (b) displayed more prominent OPN expression than hepatocellular adenoma (a). **P < 0.01 or ***P < 0.001 versus non-tumor tissue samples.

A histological analysis at 36 weeks after DEN injection showed a significantly lower prevalence of liver tumors in the OPN KO mice (14.3%) than in the WT mice (61.5%; *P*<0.05), and two of the eight tumor-bearing WT mice exhibited HCC (15.4%). Microscopically, the neoplastic nodules from the OPN KO mice at 36 weeks were sharply demarcated and consisted of well-differentiated neoplastic cells exhibiting trabecular patterns (Figure 1A). WT mice at 36 weeks showed similar histologic pattern but larger neoplastic nodules bulging from the capsular surface (Figure 1A). Meanwhile, HCC from WT mice at 36 weeks consisted of poorly differentiated neoplastic cells showing solid growth pattern with irregular border (Figure 1A). In contrast, OPN KO mice at 26 weeks after DEN injection showed a 13.3% incidence rate of hepatocellular adenoma, whereas WT mice did not have liver tumors at this time point, although this tendency was not found to be statistically significant. The neoplastic nodules from the OPN KO mice at 26 weeks showed similar histopathological features to nodules from the OPN KO mice at 36 weeks (Figure 1A).

### OPN expression is increased in human HCC tissue samples

Based on our results, we performed IHC for OPN in tumor-bearing WT mice, and six of the eight WT mice demonstrated a significantly higher degree of OPN expression in the cytoplasm of tumor cells compared to adjacent normal areas (*P*<0.01; Figure 1Ba–1b and 1C). Although this observation was based on only two cases, the degree of OPN expression was stronger in carcinomas than in adenomas (Figure 1Ba–1b).

We also carried out IHC for OPN in eight cases of human HCC. Similarly to the mouse results, seven of the eight cases showed higher OPN expression (Figure 1Bc–1d). Tumor tissue from those seven cases showed diffuse and moderate to strong OPN expression in the cytoplasm of tumor cells, whereas OPN expression was rarely or weakly observed in the paired non-tumor tissue (*P*<0.001; Figure 1Bc–d and 1C). In particular, tumor cells located on the boundary of tumor tissue prominently expressed OPN in all eight cases (Figure 1Bd).

### OPN depletion promotes apoptotic cell death in mouse liver

**Figure 2:**
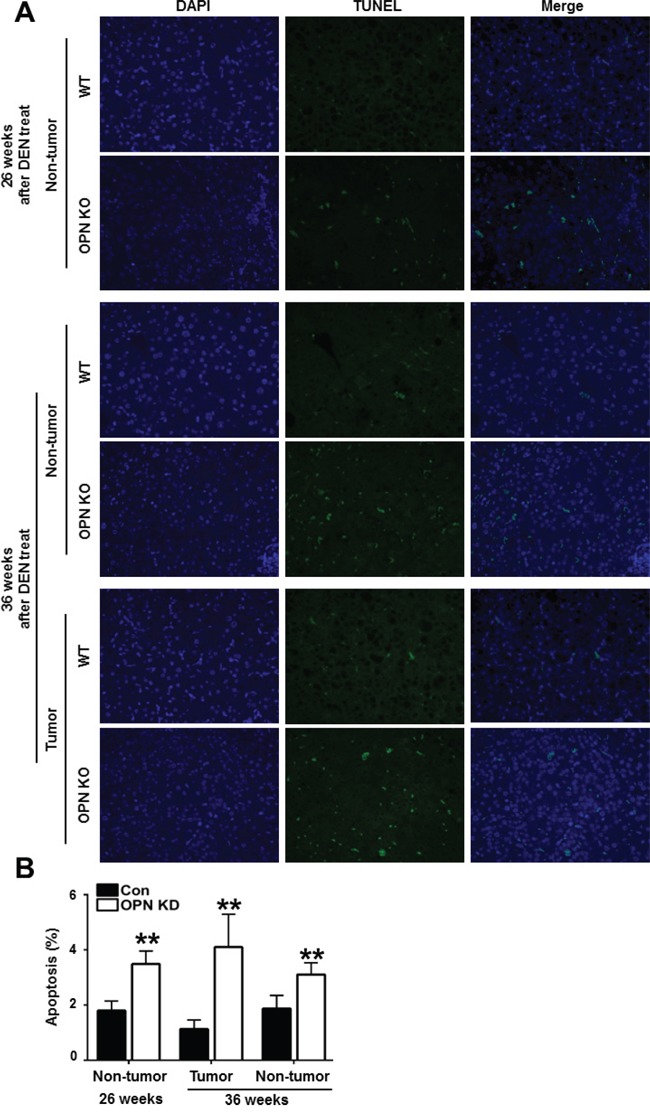
Apoptotic cell death in mouse liver tissue samples **A**. Representative photomicrographs of total cells and TUNEL-positive cells in the liver tissue of DEN-treated-WT and OPN KO mice. The apoptotic index in non-tumor tissue of OPN KO mice at 26 weeks was considerably higher than that observed in WT mice. At 36 weeks, OPN KO mice also showed significantly larger numbers of apoptotic cells in non-tumor and tumor tissue samples than WT mice. **B**. Results are presented as means ± SEMs (n=3–4 for non-tumor tissue from WT and OPN KO mice, n=3–4 for tumor tissue from WT mice and n=2 for tumor tissue from OPN KO mice). **P < 0.01 versus WT mice.

Next, we characterized the effect of OPN on apoptotic cell death in mouse liver tissue. Compared with WT mice at 26 weeks after DEN injection, non-tumor tissue from OPN KO mice showed a greater number of TUNEL-positive apoptotic hepatocytes (Figure 2A and 2B). In tumor tissue from mice at 36 weeks after DEN injection, the apoptotic index was considerably higher in the OPN KO mice than WT mice (Figure 2A and 2B). The extent of apoptotic cell death in non-tumor tissue from OPN KO mice was also greater than in the corresponding samples obtained from WT mice (Figure 2A and 2B). These results suggest that increased apoptosis in normal hepatocytes and in tumor cells of OPN KO mice may inhibit hepatic carcinogenesis.

### OPN increases cell viability through the inhibition of apoptotic cell death

**Figure 3:**
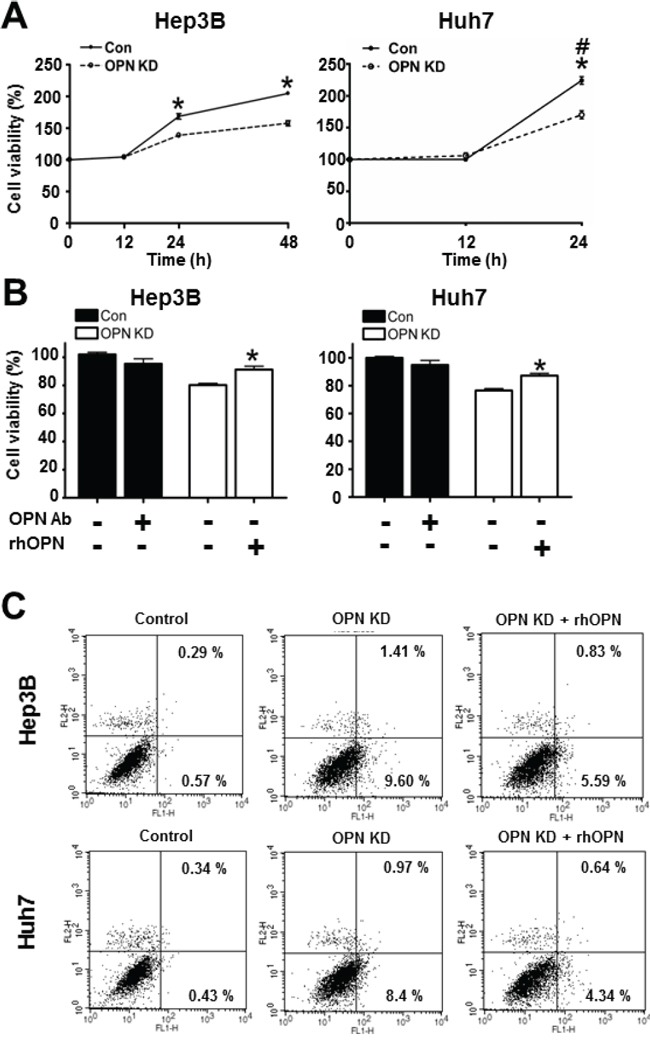
Effect of OPN downregulation on cell viability **A**. The cell viability of Hep3B and Huh7 at each time point. OPN KD Hep3B and Huh7 at 24 hours showed lower cell viability than control cells, and the cell viability of Hep3B was also decreased by OPN suppression at 48 hours. # The cell viability of Huh7 at 48 hours was not assessed due to saturation. Results are presented as SEMs (n=6 for each time point, three independent experiments). **B**. Changes in cell viability according to treatment with OPN antibody (Ab) or recombinant human OPN (rhOPN). The blockade of secreted OPN by OPN Ab in control cells caused a decrease of cell viability, although statistical significance was not observed. Supplemental rhOPN in OPN KD Hep3B and Huh7 considerably increased cell viability in both cell lines. The results are presented as means ± SEMs (n=6 for each condition, three independent experiments). **C**. Assessment of apoptotic cell death. Early and late apoptosis (lower and upper right quadrants) were more frequently observed in OPN KD Hep3B and Huh7. Supplemental rhOPN in OPN KD Hep3B and Huh7 caused a decrease in apoptosis.

As observed in mouse liver tissue, OPN is hypothesized to have a negative effect on apoptotic cell death. In order to investigate the effect of OPN on cell viability and apoptosis in human HCC, we compared growth rates between control and OPN KD Hep3B and Huh7. After incubation for 24 hours, the OPN KD Hep3B and Huh7 showed a lower number of cells than the control cells did, and the growth rate differential was more prominent at 48 hours in the Hep3B cells (Figure 3A).

**Figure 4:**
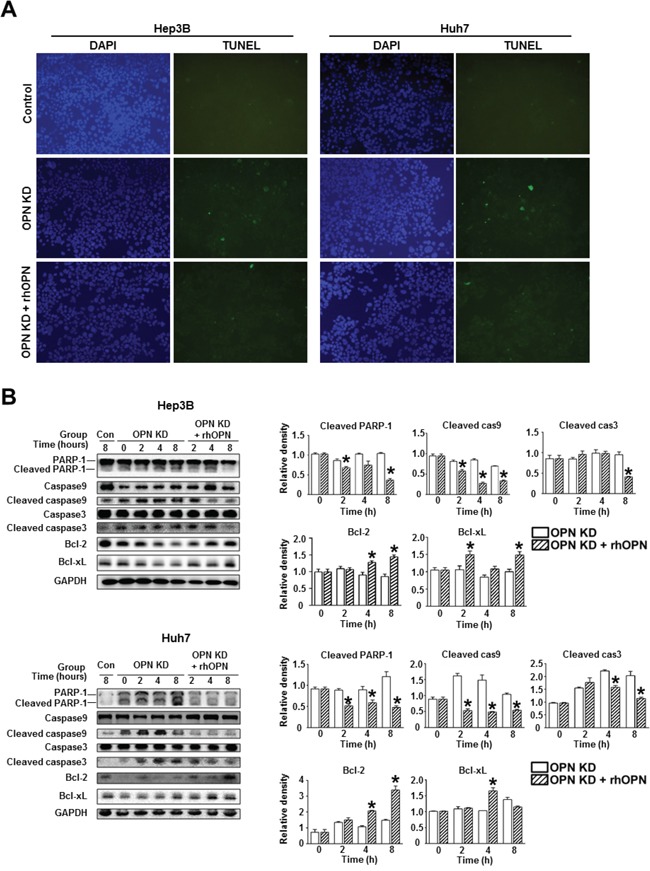
Changes in apoptosis in relation to OPN suppression **A**. TUNEL assay of the Hep3B and Huh7. OPN KD Hep3B and Huh7 showed a higher frequency of TUNEL-positive cells than control cells, and rhOPN treatment of OPN KD Hep3B and Huh7 reduced apoptotic cell death. **B**. Western blotting for the pro-apoptotic proteins PARP-1, caspase 9, and caspase 3, and the anti-apoptotic proteins Bcl-2 and Bcl-xL. The relative expression levels of apoptosis-related proteins in Hep3B (upper panels) and Huh7 (lower panels). The cleavage of PARP-1, caspase 9, and caspase 3 in OPN KD Hep3B and Huh7 from both cell lines was suppressed by supplementation with rhOPN, while the expression of anti-apoptotic proteins was upregulated. The results are presented as means ± SEMs (n=3 for each condition). * *P* < 0.05 versus OPN KD Hep3B and Huh7 under the corresponding culture conditions.

Furthermore, rhOPN treatment of OPN KD Hep3B and Huh7 at 12 hours after seeding restored cell viability to a similar degree as observed in control cells (Figure 3B). Accordingly, treatment of OPN antibody on control Hep3B and Huh7 decreased cell viability, but not to a significant extent (Figure 3B). In order to clarify the role of OPN in cell viability, we also carried out an annexin V assay and a TUNEL assay. The increased proportion of early apoptotic cells in OPN KD Hep3B and Huh7 diminished in rhOPN-treated OPN KD Hep3B and Huh7 (Figure 3C). The TUNEL assay results also indicated that rhOPN treatment reduced apoptotic cell death in OPN KD Hep3B and Huh7 (Figure 4A). However, supplemental OPN antibody to control Hep3B and Huh7 had relatively minimal effect on early and late apoptosis in the annexin V assay (Figure S2A)

Consistent with the TUNEL assay results, OPN KD Hep3B and Huh7 showed lower expression levels of anti-apoptotic proteins (Bcl-xL and Bcl-2) and higher expression levels of pro-apoptotic proteins (cleaved PARP-1, caspase 9, and caspase 3) than control cells (Figure 4B). However, the expression levels of these proteins in the OPN KD Hep3B and Huh7 were similar to those observed in control cells following rhOPN treatment (Figure 4B). Although the changes of apoptotic cell death in the annexin V assay were not remarkable, the expression levels of pro-apoptotic proteins (cleaved PARP-1 and caspase 3) in Hep3B and Huh7 were increased by the incubation with the OPN antibody (Figure S2B).

### OPN upregulates EGFR expression and related signaling pathways

**Figure 5:**
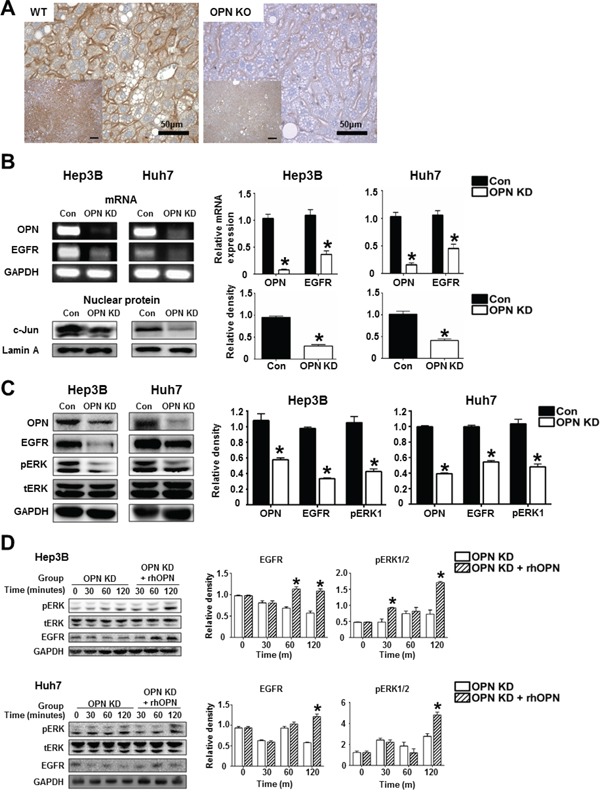
The regulatory effect of OPN on EGFR expression **A**. Representative photomicrographs of immunohistochemistry for EGFR in liver tissue samples of WT and OPN KO mice at 36 weeks after DEN injection. EGFR expression in the cellular membrane of hepatocytes was stronger in WT mice than in OPN KO mice. *Bar* = 400 μm (inserts) **B**. RT-PCR and western blot for EGFR and c-Jun. OPN suppression caused a decrease in EGFR transcription, accompanied by the downregulation of nuclear c-Jun expression in the Hep3B and Huh7. The results are presented as SEMs (n=4). * *P* < 0.05 versus control cells. **C**. Western blot for EGFR and ERK. OPN KD cells Hep3B and Huh7 showed lower expression levels of EGFR and phosphorylated ERK1. The results are presented as means ± SEMs (n=4). * *P* < 0.05 versus control Hep3B and Huh7. **D**. Western blot for EGFR and ERK according to supplementation with rhOPN in OPN KD Hep3B and Huh7. The expression of EGFR and phosphorylated ERK was increased by rhOPN treatment in a time-dependent manner. The results are presented as means ± SEMs (n=3 for each condition). * *P* < 0.05 versus OPN KD Hep3B and Huh7.

We then investigated the mRNA and protein levels of EGFR and related molecules depending on OPN expression. IHC demonstrated that WT mice at 36 weeks after DEN injection showed extensive EGFR expression in the cellular membranes, while weakly positive expression was observed in OPN KO mice (Figure 5A). *In vitro*, the mRNA level of EGFR and the protein level of nuclear c-Jun were significantly decreased by OPN suppression in the Hep3B and Huh7 (Figure 5B). Likewise, EGFR, phosphorylated ERK1 expression in OPN KD Hep3B and Huh7 were lower than was observed in control cells (Figure 5C). The expression levels of EGFR and phosphorylated ERK in OPN KD Hep3B and Huh7 were increased by supplemental rhOPN in a time-dependent manner (Figure 5D).

### OPN expression is positively correlated with EGFR expression in human HCC tissue

**Figure 6:**
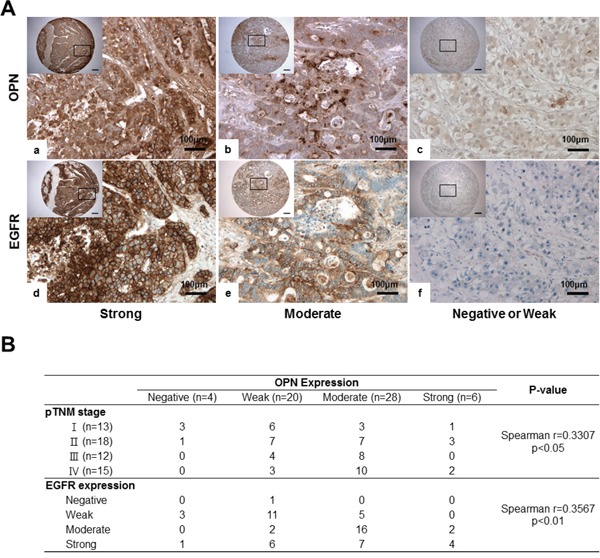
IHC for OPN and EGFR in human HCC tissue samples **A**. Representative photomicrographs of IHC for OPN and EGFR. Serially sectioned human HCC samples showing strong (a, d), moderate (b, e) and weak expression of OPN and EGFR (c, f). *Bar* = 400 μm (inserts). **B**. Correlations among OPN expression, pTNM, stages and EGFR expression.

EGFR expression showed a significant positive correlation with OPN expression in HCC (*P*<0.01, r=0.3567; Figure 6B). In cases where OPN expression was moderate or strongly positive, 85.3% (29/34) exhibited moderately or strongly positive expression of EGFR (Figure 6A and 6B). Conversely, in the 24 cases showing negative or weakly positive expression of OPN, negative or weakly positive expression of EGFR was observed in 15 cases (62.5%) (Figure 6A and 6B).

We also found that the degree of OPN expression was correlated with the pTNM stage (*P*<0.05; Figure 6B). More than half of the samples displayed a negative or weakly positive expression of OPN in stage I or II patients (Figure 6B); however, moderate to strongly positive expression of OPN was observed in 66.7% and 80% of stage III and IV samples, respectively (Figure 6B).

## DISCUSSION

In the past decades, OPN has been found to play an important role in oncogenic processes contributing to HCC and liver cirrhosis [22, 23]. Huang *et al*. showed that plasma OPN levels were positively correlated with hepatitis C virus infection and the grades of hepatic inflammation and fibrosis in human patients [24]. A previous study found that nonalcoholic steatohepatitis-related cirrhosis was increased by Hedgehog pathway-mediated OPN overexpression [25]. In the present study, we focused on the effect of OPN on the development of chemically induced HCC and the underlying mechanism involving apoptosis. We showed that hepatic carcinogenesis was considerably inhibited by OPN deficiency at 36 weeks, accompanied by the increase of apoptotic cell death in OPN KO mice. Meanwhile, OPN KO mice at 26 weeks showed the development of hepatocellular adenoma in 2/15 mice, whereas WT mice had no tumors; there were no statistical significance. In comparison with the incidence of tumor at 26 weeks, no increase of tumor incidence at 36 weeks was observed in OPN KO mice, 8/13 WT mice had hepatocellular adenoma and carcinoma. Based on these results, tumor development and progression worsened by OPN. OPN expression was prominent in the tumor tissue of WT mice, whereas adjacent liver tissue rarely showed OPN expression. Similarly, strong OPN expression was noted in most of human HCC samples. These results correspond to previous clinical studies that found OPN overexpression to be positively correlated with tumor progression.

Apoptosis is an essential process for maintaining homeostasis in normal tissue, and is connected with carcinogenesis because tumor cells may be able to evade apoptotic stimuli [26]. In a previous study, we suggested that OPN protected gastric epithelial cells and cancer cells from inducible nitric oxide synthase-mediated apoptosis through STAT1 downregulation, thereby promoting the development and progression of gastric cancer [27]. Likewise, some studies have reported that OPN played an important role in inhibiting apoptosis [28, 29]. In a mouse model, Hsieh *et al*. found that OPN deficiency promoted apoptosis and delayed the development of squamous papilloma [30]. In addition, it has been demonstrated through IHC that the anti-apoptotic effect of OPN may be related to the regulation of nuclear factor-kappa B (NF-κB) expression in human renal cell carcinoma [31]. In accordance with these results, we demonstrated that the tumor tissue samples and non-tumor tissue samples of OPN KO mice displayed a higher apoptotic index than was observed in WT mice, and that OPN suppression in human HCC cells also promoted apoptotic cell death. Previous studies have shown that OPN has two isoforms: a secreted form (sOPN) and an intracellular form (iOPN), generated by alternative translation [32]. Based on the observation that the supplemental provision of rhOPN to OPN KD human HCC cells inhibited apoptosis, and that OPN overexpression in cells rarely affect cell viability and apoptotic cell death in the present study, it may be hypothesized that the anti-apoptotic effect of OPN is due to sOPN rather than iOPN (Figure S3A and SB). Some reports support the possibility that the role of OPN in apoptotic cell death is mediated by interactions between OPN and its surface receptors with related downstream signaling. Zhao *et al*. previously found that OPN downregulation led to the inhibition of integrin expression, which could block the activity of NF-κB [33]. In addition, binding to the CD44 variants was able to exert significant suppressive effects on apoptosis of tumor cells through the phosphoinositide 3-kinase/Akt pathway [34]. Our data suggest that the increased apoptosis of hepatocytes and tumor cells may cause the suppression of hepatic carcinogenesis in OPN KO mice.

Next, we evaluated EGFR expression according to OPN regulation in order to explore the underlying mechanism through which OPN suppresses apoptosis. It has been confirmed that EGFR and downstream signaling significantly contribute to the carcinogenesis of various epithelial cancers [35, 36]. Similarly, EGFR blockade dramatically enhanced the apoptosis of cancer cells induced by ultraviolet radiation and chemotherapeutic agents [37]. In human cancer, sustained EGFR activation is commonly observed due to EGFR overexpression or the mutation of EGFR, leading to various tumor-promoting activities, including the inhibition of apoptosis [38, 39]. This anti-apoptotic effect of EGFR is mediated by the MAPK/ERK kinase (MEK)/MAPK signaling pathway in many types of normal cells as well as cancer cells [40, 41]. In agreement with previous studies, we found that WT mice showing a high EGFR expression level presented a lower apoptotic index in comparison to OPN KO mice, and that OPN suppression led to decreased EGFR expression and phosphorylation of ERK, as well as increased apoptotic cell death in human HCC cells. The activity of EGFR can be regulated ligand-dependently or transcriptionally, and the latter is mediated by promoter-binding factor [14, 38]. Zenz *et al*. previously demonstrated that c-Jun, a member of the activator protein 1 family, played a crucial role in the transcriptional regulation of EGFR expression in a c-Jun conditional KO mice model [41]. They also showed that the lack of c-Jun ultimately led to apoptosis in keratinocytes. Based on this previous finding, we investigated the expression level of c-Jun depending upon OPN regulation, and found that c-Jun expression was suppressed by shRNA-mediated OPN KD. Additionally, it may have been the case that c-Jun-mediated EGFR expression was regulated by secreted OPN, since supplemental rhOPN in OPN KD human HCC cells (not OPN overexpression) led to upregulated EGFR expression (Figure S3B). In combination with previous studies, our findings suggest that OPN is an important factor for inducing c-Jun-mediated EGFR transcription, resulting in the inhibition of apoptotic cell death.

In conclusion, we showed that OPN deficiency inhibited DEN-induced hepatic carcinogenesis in a mouse model, which could be linked with increased apoptotic cell death. In addition, *in vitro* study suggested the possibility that the anti-apoptotic effect of OPN may be related to the transcriptional upregulation of EGFR and the activation of the downstream molecule, p-ERK, which is mediated by c-Jun. Likewise, a positive correlation between OPN and EGFR expression was also identified in human HCC tissue as shown in a previous study [42]. In contrast to apoptosis, OPN suppression did not affect cell proliferation significantly (Figure S1). OPN and related signaling pathways are also involved in cell proliferation, invasion, and metastasis. Yoo *et al*. and Zhao *et al*. have previously described these roles of OPN in HCC using an *in vitro* and xenograft model [21, 33]. On the other hand, Fan *et al*. proposed that OPN deficiency aggravates DEN-induced hepatic carcinogenesis based on the size and multiplicity of liver tumor. It was a new perspective in the roles of OPN in carcinogenesis [43]. While they mainly focused on the roles of OPN in tumor progression because liver tumors developed in all mice at 36 weeks after the injection of DEN, our mouse study could more logically explain the effect of OPN deficiency on tumor development considering many previous studies on the tumor-promoting effects of OPN. In the present study, we focused on the role of OPN in hepatocyte and cancer cells based on the histopathologic findings and IHC results from mouse and human HCC samples. However, further studies using liver-specific OPN KO mice rather than whole tissue-OPN KO mice or a comparison of these two types of OPN KO mice could be useful for determining the role of OPN in hepatic carcinogenesis in greater detail because human HCC can be closely related to immune response against to cellular damage and infectious agents. Despite some limitations of this study, our findings suggest that OPN directly contributes to the development of HCC, which is positively correlated with the EGFR-mediated anti-apoptotic effect. Therefore, the induction of apoptosis in cancer cells through targeting OPN and EGFR may be a helpful approach for the prevention and treatment of human HCC.

## MATERIALS AND METHODS

### Induction of HCC in mice

Male C57BL/6-Spp1^tm1Blh^ (OPN^-/-^) (OPN KO) mice, purchased from Jackson Laboratory (Bar Harbor, ME, USA), and WT mice were divided into four groups (26 and 36 weeks with either OPN KO or WT) with seven mice in each group as controls. The mice at 2 weeks old were injected with 25 mg/kg of DEN (Sigma Chemical Co., St. Louis, MO, USA) or vehicle intraperitoneally once to induce hepatic carcinogenesis. Mice in the control groups were not subjected to DEN injection. All mice were sacrificed 26 and 36 weeks after the injection of DEN. This study was approved by the Institutional Animal Care and Use Committee of Seoul National University, and all experiments were performed according to the Guide for Care and Use of Laboratory Animals published by the Institute for Laboratory Animal Research (Washington, DC, USA).

### Immunohistochemical staining for OPN and TUNEL assay

In order to perform immunohistochemical staining for OPN in human and mouse tissues, replicate sections of paraffin-embedded liver tissue were mounted on silicon-coated slides, dewaxed, and rehydrated, and antigen retrieval was then performed. The slides were incubated with anti-mouse or-human OPN antibody (1:100; R&D Systems, Minneapolis, MN, USA). Quantitation of immunoreactivity was performed using an H-scoring system, in which scores were calculated based on the intensity and number of positive cells according to the equation: Score = (3 × % intensely positive) + (2 × % moderately positive) + (1 × % weakly positive).

Apoptotic cell death in mouse tissue samples and Hep3B and Huh7 was determined through the terminal deoxynucleotidyl transferase dUTP nick-end labeling (TUNEL) assay using the Fluorecein FragEL DNA Fragmentation Detection kit (Calbiochem, Darmstadt, Germany).

### Analysis of cell viability and flow cytometry for the apoptosis

To identify the effect of OPN on cell viability, a trypan blue exclusion assay was performed. After seeding on six-well plates at 1 × 10^5^ cells/well and incubation for 12, 24, and 48 hours with 1% fetal bovine serum (FBS), control and short-hairpin RNA (shRNA)-mediated OPN knockdown (KD) Hep3B and Huh7 cells were harvested, stained with trypan blue solution, and then counted using an inverted microscope.

Next, we performed anti-human OPN antibody (R&D Systems) or recombinant human OPN (rhOPN; R&D Systems) treatment on control and OPN KD Hep3B and Huh7 to investigate the effect of secreted OPN on cell viability. Before incubation with rhOPN or anti-human OPN antibody, the minimal cytotoxic concentrations were determined. After seeding on six-well plates at 1 × 10^5^ cells/well for 12 hours with 1% FBS, control Hep3B and Huh7 were incubated with 1 μg/mL of anti-human OPN antibody, and OPN KD Hep3B and Huh7 were incubated with 2 μg/mL of rhOPN for 12 hours. These cells were subjected to the trypan blue exclusion assay after being harvested.

For analysis of the apoptosis, control and OPN KD Hep3B and Huh7 treated with 2 μg/mL of rhOPN from the Hep3B and Huh7 lines were cultured at 2 × 10^5^ cells/well for 24 hours with 1% FBS, and harvested, washed, and re-suspended with cold phosphate-buffered saline. Apoptotic cell death was determined using an Annexin V Apoptosis Detection Kit FITC (eBioscience, San Diego, CA, USA). Cell-associated fluorescence was measured using a FACSCalibur apparatus (BD Bioscience). We also performed a TUNEL assay and a western blot for apoptosis-related proteins. After seeding on Lab-Tek Chamber slides (Thermo Fisher Scientific, Hudson, NH) at 5 × 10^4^ cells/well and incubation with 2 μg/mL of rhOPN for 24 hours, the TUNEL assay was performed on control, non-treated OPN KD and rhOPN-treated OPN KD Hep3B and Huh7. Similarly, control and OPN KD Hep3B and Huh7 incubated with 2 μg/mL of rhOPN from both cells were harvested and lysed at appropriate time points for western blotting.

### Tissue microarray-based immunohistochemical staining of OPN and EGFR in human HCC tissue samples

Tissue-microarray (TMA) slides containing 58 HCC samples were purchased from SuperBioChips Laboratories (Seoul, Korea, www.tissue-array.com). Immunohistochemistry (IHC) for OPN and EGFR on serially sectioned TMA slides was carried out using the BOND-MAX automated immunostainer (Leica Microsystems, Bannockburn, IL, USA) with the Bond Polymer Refine detection kit (Leica). Anti-OPN antibody (1:50; R&D Systems) or anti-EGFR antibody (1:100; Ventana Medical Systems, Oro Valley, AZ, USA) were employed as the primary antibody. Each stain was assessed according to the intensity (negative, 0; weak, 1; moderate, 2; intense, 3) and area (none, 0; focal, 1; multifocal, 2; diffuse, 3) of positive cells. The overall grade of each stain was obtained by multiplying the area score by the intensity score (negative, 0; weak, 1 or 2; moderate, 3 or 4; intense, 6 or 9).

### Statistical analysis

All data were expressed as means ± standard errors (SEMs). Statistical analyses were performed using GraphPadPrism6 (version 6.0; GraphPad Software, San Diego, CA, USA). OPN expression in tumor tissue and non-tumor tissue samples from mouse and human liver was compared using the paired two-tailed Student's *t*-test. The relationship between the OPN genotype and the incidence of hepatocellular adenoma or carcinoma was analyzed using the chi-square test. Correlations between OPN expression and clinicopathological features or EGFR expression were analyzed using Spearman's correlation test. Other data were analyzed using the unpaired two-tailed Student's *t*-test. *P*-values <0.05 were considered to indicate statistical significance.

Detailed Materials & Methods are described in Supplementary Data.

## SUPPLEMENTARY FIGURES


