# Supplementary material for: Suppression of osteopontin inhibits chemically induced hepatic carcinogenesis by induction of apoptosis in mice
Source: Oncotarget. 2016 Nov 23;7(52):87219–31. doi: 10.18632/oncotarget.13529 (PMC5349983; doi:10.18632/oncotarget.13529)
Supplement: Supplementary file 1 [file oncotarget-07-87219-s001.pdf]

Suppression of osteopontin inhibits chemically induced hepatic carcinogenesis by induction of apoptosis in mice

SUPPLEMENTARY DATA

Gross and histopathological examinations

The mice were sacrificed after 24 hours of fasting. The liver was excised from each mouse and the number and size of nodules were measured. All liver tissue samples containing adjacent normal tissue were fixed in neutral-buffered 10% formalin. After 24 hours of fixation, the liver tissue samples were processed using a routine method, embedded in paraffin, and stained with hematoxylin and eosin for diagnosis.

Immunohistochemical staining for OPN and TUNEL assay

In order to perform immunohistochemical staining for OPN in human and mouse tissue, replicate sections of paraffin-embedded liver tissue were mounted on silicon-coated slides, dewaxed, and rehydrated, and antigen retrieval was then performed by heating at 100°C for 20 minutes in a 0.01 M citrate buffer (pH 6.0). The inactivation of endogenous peroxidase and blocking of non-specific protein binding were relieved utilizing hydrogen peroxide and serum-free protein block solution (DakoCytomation, Glostrup, Denmark). The slides were incubated with anti-mouse or-human OPN antibody (1:100; R&D Systems, Minneapolis, MN, USA). After incubation with the primary antibody, slides were stained using the indirect labeling streptavidin avidin-biotin technique with 3-3'-diaminobenzene as a substrate.

We also performed IHC for proliferating cell nuclear antigen (PCNA) using anti-PCNA antibody (1:100; Santa Cruz Biotechnology, Santa Cruz, CA) in order to investigate whether OPN depletion affected cell proliferation.

Quantitation of immunoreactivity was performed using an H-scoring system, in which scores were calculated based on the intensity and number of positive cells according to the equation: Score = (3 × % intensely positive) + (2 × % moderately positive) + (1 × % weakly positive).

Apoptotic cell death in mouse tissue samples and human HCC cell lines was determined through the terminal deoxynucleotidyl transferase dUTP nick-end labeling (TUNEL) assay using the Fluorecein FragEL DNA Fragmentation Detection kit (Calbiochem, Darmstadt, Germany).

Generation of OPN knockdown cell lines

The human HCC cell lines Hep3B and Huh7 were purchased from the Korean Cell Line Bank (KCLB; Seoul, Korea). Hep3B and Huh7 cells were separately cultured in Dulbecco's modified Eagle medium (Gibco, Grand Island, NY, USA) or RPMI-1640 medium (Gibco) containing 10% fetal bovine serum (FBS; Gibco), 1% penicillin, and streptomycin (Invitrogen Biotechnology, Grand Island, NY, USA). For the stable knockdown of the Spp1 gene in the Hep3B and Huh7 cell lines, a lentiviral vector-mediated short-hairpin RNA (shRNA) construct targeting the human Spp1 gene (Sigma-Aldrich, St. Louis, MO, USA) with the pLKO.1-puro enhanced green fluorescent protein control vector (Sigma) were produced from accession number NM_000582. The lentivirus was generated by the cotransfection of the shRNA-expressing vector and packaging vectors (Addgene) into 293Ts cell through Lipofectamine™ 2000 (Invitrogen Biotechnology). After transfection for 48 hours, virus-containing supernatant was collected, filtered using a 0.45-µm filter, assessed for titer values, and used for viral transduction with 10 µg/mL of polybrene. Cells were selected over the course of three days through treatment with 2 µg/mL of puromycin after viral transduction. Knockdown efficiency and OPN-related molecular changes were confirmed by quantitative real-time reverse transcription polymerase chain reaction (RT-PCR) and western blotting.

OPN transfection

cDNA containing the coding region of human OPN-a, which was subcloned into the expression vector pDest-490, was a gift from Xin Wang (Addgene plasmid # 17590) [1]. Hep3B or Huh7 cells were transfected with 500 ng of pDest490-OPN-a or pDest490 control vector using Lipofectamine™ 2000 (Invitrogen Biotechnology) according to the manufacturer's instructions. At the appropriate time point after transfection, the cells were harvested to perform an Annexin V assay or a western blot analysis.

RNA extraction, RT-PCR, and quantitative real-time RT-PCR

The total RNA from Hep3B and Huh7 cells was extracted using the RNeasy Plus Mini kit (Qiagen, Hilden, Germany), and 500 ng of total RNA from each

sample was reverse-transcribed using the QuantiTect Reverse Transcription kit (Qiagen) and analyzed by quantitative real-time RT-PCR using the Rotor-Gene SYBR Green PCR kit (Qiagen) with specific primers. The amplification and quantitation of target genes were performed using the Rotor-Gene Q and the manufacturer-provided software (Qiagen). The amount of the target gene was calculated using mRNA encoding glyceraldehyde 3-phosphate dehydrogenase (GAPDH) as a housekeeping gene. The amplified target genes were also loaded on 2% agarose gel. The primer pairs for human genes were F: 5'-GCC AAG GCA CGA GTA ACA AGC-3' and R: 5'-AGG GCA ATG AGG ACA TAA CC-3' for EGFR, F: 5'-TGA AAC GAG TCA GCT GGA TGA CCA-3' and R: 5'-GCT CTC ATC ATT GGC TTT CCG CTT-3' for OPN and F: 5'-GAG TCA ACG GAT TTG GTC G-3' and R: 5'-TGG AAT CAT ATT GGA ACA TGT AAA C-3' for GAPDH.

Western blotting

The total protein of the cells was extracted using the RIPA II Cell Lysis Buffer (GenDEPOT, Barker, TX, USA) supplemented with a protease inhibitor cocktail (GenDEPOT) and phosphatase inhibitor cocktail (GenDEPOT). The cell fraction for extracting nuclear proteins was performed using the Qproteome Cell Compartment Kit (Qiagen). Equal amounts of protein (25 µg) from cell pellets were loaded by sodium dodecyl sulfate-polyacrylamide gel electrophoresis (SDS-PAGE) on 10% or 12% gels and transferred onto 0.2-µm nitrocellulose membranes (GE Healthcare, Buckinghamshire, UK). Proteins were detected with one of the following antibodies: anti-human OPN (1:500; R&D Systems), anti-EGFR (1:1000; Santa Cruz Biotechnology), anti-ERK1/2 (1:1000; Cell Signaling Technology, Danvers, MA), anti-phosphorylated ERK1/2 (1:1000; Cell Signaling Technology), anti-caspase 3 (1:1000; Santa Cruz Biotechnology), anti-caspase9 (1:1000; Santa Cruz Biotechnology), anti-Bcl-2 (1:1000; Cell Signaling Technology), anti-Bcl-xL (1:1000; Cell Signaling Technology), anti-c-Jun (1:500; Santa Cruz Biotechnology), anti-lamin A (1:1000; Abcam, Cambridge, MA), or anti-PARP-1 (1:1000; Santa Cruz Biotechnology) antibody. The membranes were then incubated with horseradish peroxidase-conjugated rabbit anti-goat IgG antibody (GenDEPOT) or goat anti-rabbit IgG antibody (Millipore, Billerica, MA, USA), as appropriate. GAPDH (Cell Signaling Technology) was used as an internal control.

REFERENCE

1. Takafuji V, Forgues M, Unsworth E, Goldsmith P, Wang X. An osteopontin fragment is essential for tumor cell invasion in hepatocellular carcinoma. Oncogene. 2007; 26:6361-6371.

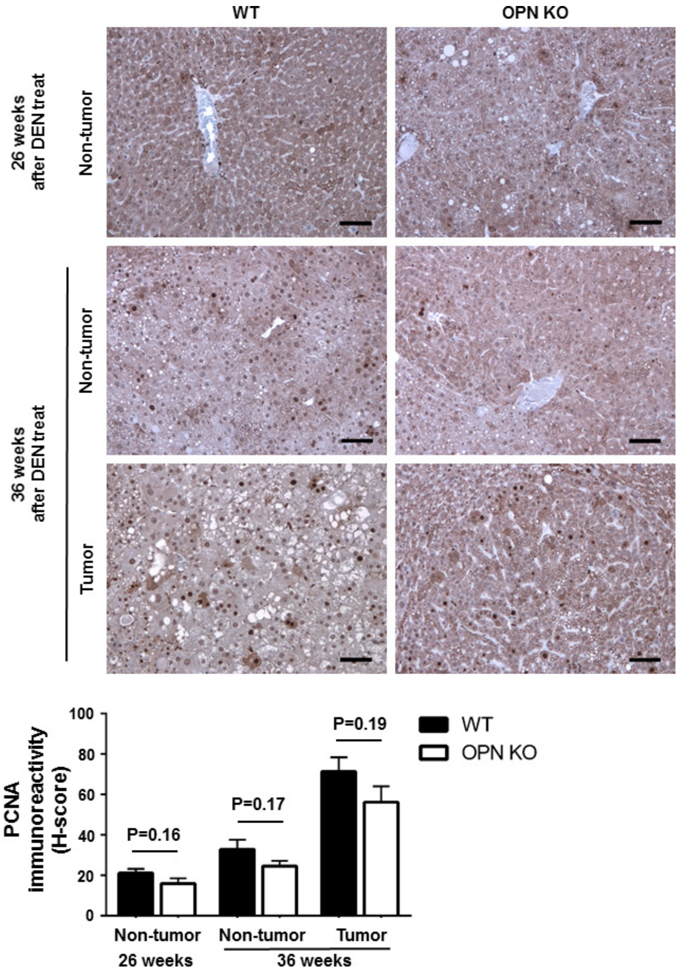

Supplementary Figure S1: IHC for proliferating cell nuclear antigen (PCNA). At 26 and 36 weeks after DEN injection, WT mice displayed more PCNA-positive hepatocytes in non-tumor and tumor tissue samples than did OPN KO mice; however, this tendency was not found to be statistically significant.

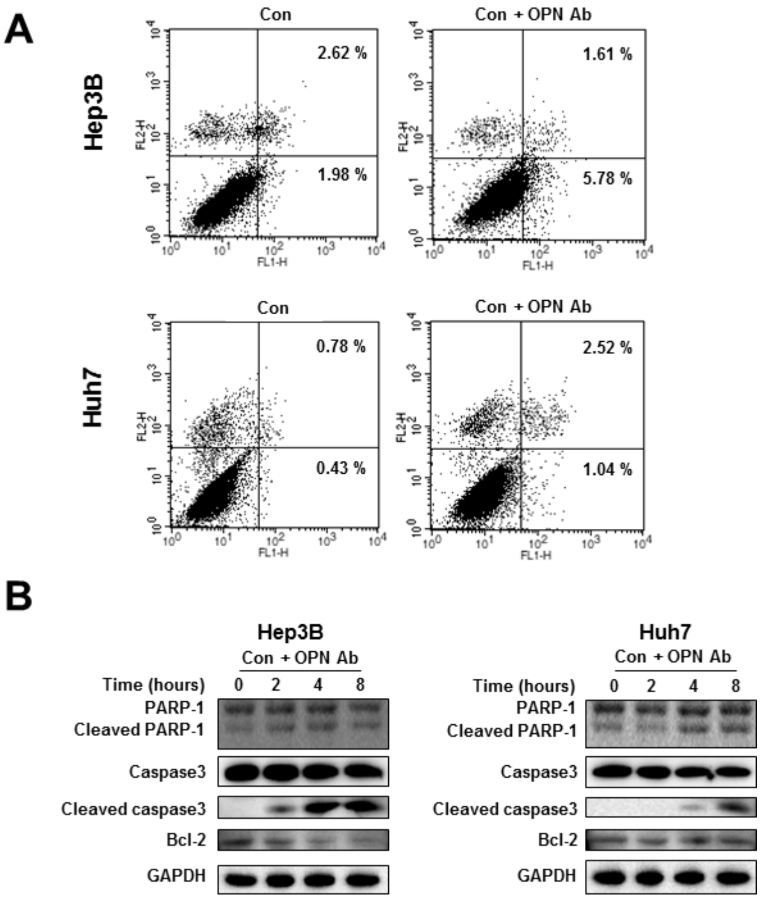

Supplementary Figure S2: Annexin V assay and western blot for apoptosis. A. There were observed that a slightly increased proportion of early or late apoptosis in Hep3B and Huh7 cells incubated with anti-OPN antibody. **B.** Control shRNA-transfected Hep3B and Huh7 cells showed increased expressions of cleaved PARP-1 and caspase3 depending on the incubation with anti-OPN antibody in a time-dependent manner.

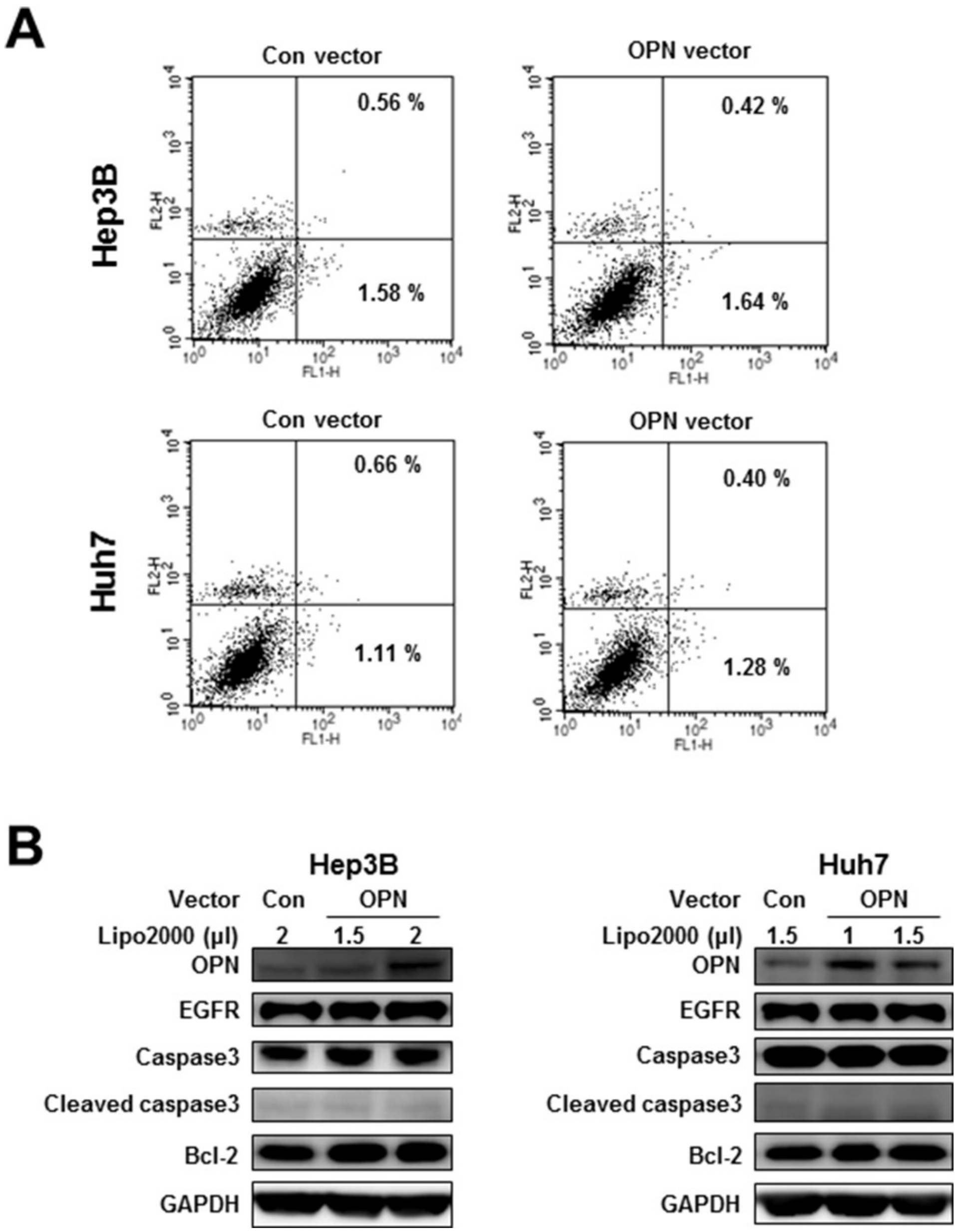

Supplementary Figure S3: Annexin V assay and western blot for apoptosis in OPN overexpression. pDest-490-OPN-a vector transfection for OPN overexpression neither cause decreased apoptotic cell death **A.** nor significant change of the expression levels of EGFR and apoptotic proteins **B.**
